# Causal relationship between polycystic ovary syndrome and chronic kidney disease: A Mendelian randomization study

**DOI:** 10.3389/fendo.2023.1120119

**Published:** 2023-03-15

**Authors:** Yufei Du, Fengao Li, Shiwei Li, Li Ding, Ming Liu

**Affiliations:** Department of Endocrinology and Metabolism, Tianjin Medical University General Hospital, Tianjin, China

**Keywords:** polycystic ovary syndrome, chronic kidney disease, Mendelian randomization, single nucleotide polymorphisms, causality

## Abstract

**Objective:**

Polycystic ovary syndrome is one of the most common endocrine disorders among women of childbearing age. The relationship between polycystic ovary syndrome and chronic kidney disease remains unclear and controversial. In this study, we investigated the causal role of polycystic ovary syndrome in the development of chronic kidney disease using the two-sample Mendelian randomization method.

**Methods:**

Public shared summary-level data was acquired from European-ancestry genome wide association studies. We finally obtained 12 single nucleotide polymorphisms as instrumental variables, which were associated with polycystic ovary syndrome in European at genome-wide significance (P < 5 × 10^−8^). Inverse-variance weighted method was employed in the Mendelian randomization analysis and multiple sensitivity analyses were implemented. Outcome data were obtained from the Open GWAS database.

**Results:**

A positive causal association was observed between polycystic ovary syndrome and chronic kidney disease (odds ratio [OR]=1.180, 95% confidence interval [CI]: 1.038-1.342; P=0.010). Further analyses clarified that causal relationship exist between polycystic ovary syndrome and some serological indicators of chronic kidney disease (fibroblast growth factor 23: OR= 1.205, 95% CI: 1.031-1.409, P=0.019; creatinine: OR= 1.012, 95% CI: 1.001-1.023, P=0.035; cystatin C: OR= 1.024, 95% CI: 1.006-1.042, P=0.009). However, there was no causal association of polycystic ovary syndrome with other factors in the data sources we employed.

**Conclusions:**

Our results indicate an important role of polycystic ovary syndrome in the development of chronic kidney disease. This study suggests that regular follow-up of renal function in patients with polycystic ovary syndrome is necessary for the early treatment of chronic kidney disease.

## Introduction

Polycystic ovary syndrome (PCOS) is one of the most common endocrinopathies, affecting 4–21% of women among premenopausal women (1–3). The features of PCOS include oligomenorrhoea, infrequent ovulation, polycystic ovarian morphology (4) and a series of metabolic disorders including hyperandrogenism, hyperinsulinemia, gonadotropin imbalance, dyslipidemia, and often accompanied by increased visceral fat (5). The etiology and pathological mechanism of this disease remains unclear. Genome-wide association studies (GWAS) provided genetic evidence for further PCOS research (6–9). As mentioned above, patients with PCOS are accompanied by a series of metabolic disorders, and whether this leads to the occurrence of other chronic diseases is still lacking sufficient evidence.

Chronic kidney disease (CKD) is a common chronic disorder that refers to the long-term loss of renal function, eventually may progress into end-stage renal disease with a high rate of mortality (10). It affects millions of patients worldwide (11). The global burden of CKD is growing year after year, and almost 10% of adults around the world are affected by some subtypes of CKD, resulting in 1.2 million deaths and 28 million patients suffer from the disease each year (12, 13). CKD can occur for many reasons, including that more common and well-researched such as diabetes, glomerulonephritis, and cystic kidney diseases, however, the precise influencing factors of CKD are still not comprehensively studied (14). Recent studies have shown that long-term metabolic disorders, which could irreversibly impair the renal function and structure, are closely related to the occurrence of CKD (15).

The various metabolic disturbances caused by PCOS might lead to increasing incidence of CKD in later life in these PCOS patients, but few studies have focused on the long-term changes in renal function in patients with PCOS. Duleba et al. found that urinary albumin excretion (UAE) can occur in patients with PCOS with cardiovascular risk factors (16). Gozukara et al. found that although the test results of renal function were in the normal ranges in patients with PCOS, the GFR, urinary albumin excretion, and serum uric acid levels were all higher in PCOS patients than that in the controls (17). In previous animal experiments, Mohadetheh Moulana found that PCOS rats had higher renal mast cells infiltration which may lead to alteration of the immunological niche and persistent damage of kidney (18). Pruett et al. demonstrated that renal SGLT2 inhibitor could decreased the fat mass, plasma leptin, and additional therapies needed to improve renal injury in PCOS rats (19). These studies provide some supportive clues for us to understand the relationship between PCOS and CKD, but no human studies have been done to our knowledge to determine whether PCOS causes the occurrence of CKD or increase the risks for renal disease.

To gain further insight into the relationship between PCOS and CKD, we employed the Mendelian randomization (MR), a novel genetic epidemiological method that uses genetic variants as instrumental variables, to determine whether an observational association between a risk factor and an outcome is consistent with a causal effect (20). The MR methods capitalizes on the presumed random assortment of genes from parents to offspring, which provides unbiased detection of causal effects, since genetic variants are less susceptible to environmental factors (21). Using the findings for PCOS provided by the extensive GWAS data, we conducted two-sample (exposure and outcome measured in different samples) MR study to evaluate the causal effects of patients with PCOS on the risk of developing CKD, and further investigate the causal effects between genetic variants that determine the variation of PCOS and CKD risk factors, serological indicators of CKD, renal tubule injury biomarkers, separately.

## Methods

### Study design

We used a two-sample MR to investigate the causal relationship between PCOS and CKD or other factors including CKD risk factors, serological indicators of CKD and renal tubule injury biomarkers in the European population. For MR analysis, we used the inverse variance weighted (IVW) analysis accounting for correlations between instrument variables and outcomes. For the sensitivity analysis, weighted-median and MR-Egger analyses were performed. Exposure data derived from a meta-analysis, and ethics approval was obtained from the relevant research ethics committee (22). Outcome data can be obtained from the Open GWAS database (http://gwas.mrcieu.au.uk). Since this study was based on the public GWAS database, ethics approval was not required (23). This study was conducted based on the Strengthening the Reporting of Observational Studies in Epidemiology using Mendelian Randomization (STROBE-MR) guideline (24).

### Instrument variable selection

The SNPs associated to PCOS were derived from a large-scale genome-wide meta-analysis of polycystic ovary syndrome (22). The PCOS cohort included 113,238 European subjects (10,074 cases and 103,164 controls). We selected single nucleotide polymorphisms (SNPs) that have been shown to be significantly associated with PCOS (14 SNPs) at a genome-wide significance level (P < 5 × 10^−8^). Linkage disequilibrium analysis was performed based on a threshold of r2 < 0.001. In this study, 13 extracted SNPs passed the test and 1 was excluded (rs853854) for being palindromic with inter mediate allele frequencies (**Supplementary Table 1**). To avoid weak instrument bias, we calculated the F-statistic. In our study, F-statistics for the instrumental variables were strong (> 10) (25). These selected SNPs must meet the following three core assumptions. Firstly, SNPs are significantly correlated with exposure which means that the SNPs can predict exposure effectively. Secondly, the SNPs have to be independent of the outcome, namely the SNPs can only affect outcome through exposure. Thirdly, the SNPs must be independent of the confounding factors associated with exposure or outcome. Studies have found that PCOS is associated with type 2 diabetes, obesity and hypertension, which may increase the risk of CKD. The above-mentioned risk factors may also be potential confounding factor between PCOS and CKD. In order to satisfy the assumptions, we conducted further analysis after taking into account confounding factors. To investigate whether PCOS associated SNPs are significantly correlated with other confounding factors at the genome-wide level, we searched the PhenoScanner database (http://www.phenoscanner.medschl.cam.ac.uk/) for these PCOS associated SNPs. We found that rs2271194 is associated with Body mass index (BMI), which is a risk factor for CKD. The remaining SNPs are not associated with common confounding factors interrelated to PCOS and CKD such as diabetes, hypertension, obesity, glomerulonephritis and polycystic kidney disease. To rule out potential confounding factors, rs2271194 was excluded and 12 SNPs were eventually included for further analyses.

### Outcome data source

The summary level dataset of CKD was retrieved from the Open GWAS database (https://gwas.mrcieu.ac.uk/), which included 216761 participants with CKD (3902 cases and 212841 controls). Summary-level statistics of the CKD risk factors were obtained from the Open GWAS database (23). we selected several well-established risk factors for CKD, including type 2 diabetes, autoimmune diseases, cystic kidney disease, obesity, and hypertension from a review of Chen et al. (26). In order to explore the relationship between PCOS and CKD serological indicators, we also obtained data from the database and selected some serological indicators that would be used in clinical practice for analysis, mainly including fibroblast growth factor 23, creatinine, cystatin C, estimated glomerular filtration rate, β2-microglobulin and urinary albumin-to-creatinine ratio. Finally, to explore whether renal tubular injury is associated with PCOS, we also obtained biomarkers of renal tubular injury from the Open GWAS database and selected a part of common biomarkers for analysis, including insulin-like growth factor-binding protein 7, epidermal growth factor, monocyte chemoattractant protein-1, tumor necrosis factor receptor 1, tumor necrosis factor receptor 2, chitinase-3-like protein 1, kidney injury molecule 1, neutrophil gelatinase-associated lipocalin, interleukin 6 and interleukin 10 from a review of Zhang et al. (27). All data for analysis are based on European population.

### Statistical analysis

We utilized 12 genetic variants as instrumental variables to estimate the causal association between PCOS and CKD and other factors. In this study, variable selection was relied on prior information. We selected outcome variables based on previous research reports to be as close to the clinical application as possible, and we also pay attention to the latest biomarkers to explore the possibility of new discoveries. In the present study, the robust methods: inverse variance weighting (IVW), MR Egger, and weighted median were employed to assess the causal relationship. IVW, the main dependent method, equates to implement a weighted linear regression of the associations between the outcome instrumental variables (IVs) and the exposure, merges the Wald ratio estimates of each IV in a meta-analysis manner (28, 29). Sensitivity analyses, including MR-Egger and weighted median. MR Egger considers the horizontal pleiotropic effects, in which the intercept may provide evidence for potential pleiotropic effects across IVs, although its estimates may be imprecise. The weighted median estimator is a method in which at least 50% of the weights are considered valid instrumental variables (30). Weighted median method allows some genetic variants are invalid, but only if at least half of them are valid instruments (31). Heterogeneity is another major concern in MR analyses, suggesting the possible concurrent presence of pleiotropy. To evaluate the heterogeneity between the IVs, IVW, MR-Egger, and Maximum likelihood methods were used. Cochran’s Q statistic was employed to quantify the heterogeneity.

## Results

### The association between PCOS and CKD

**Figure 1:**
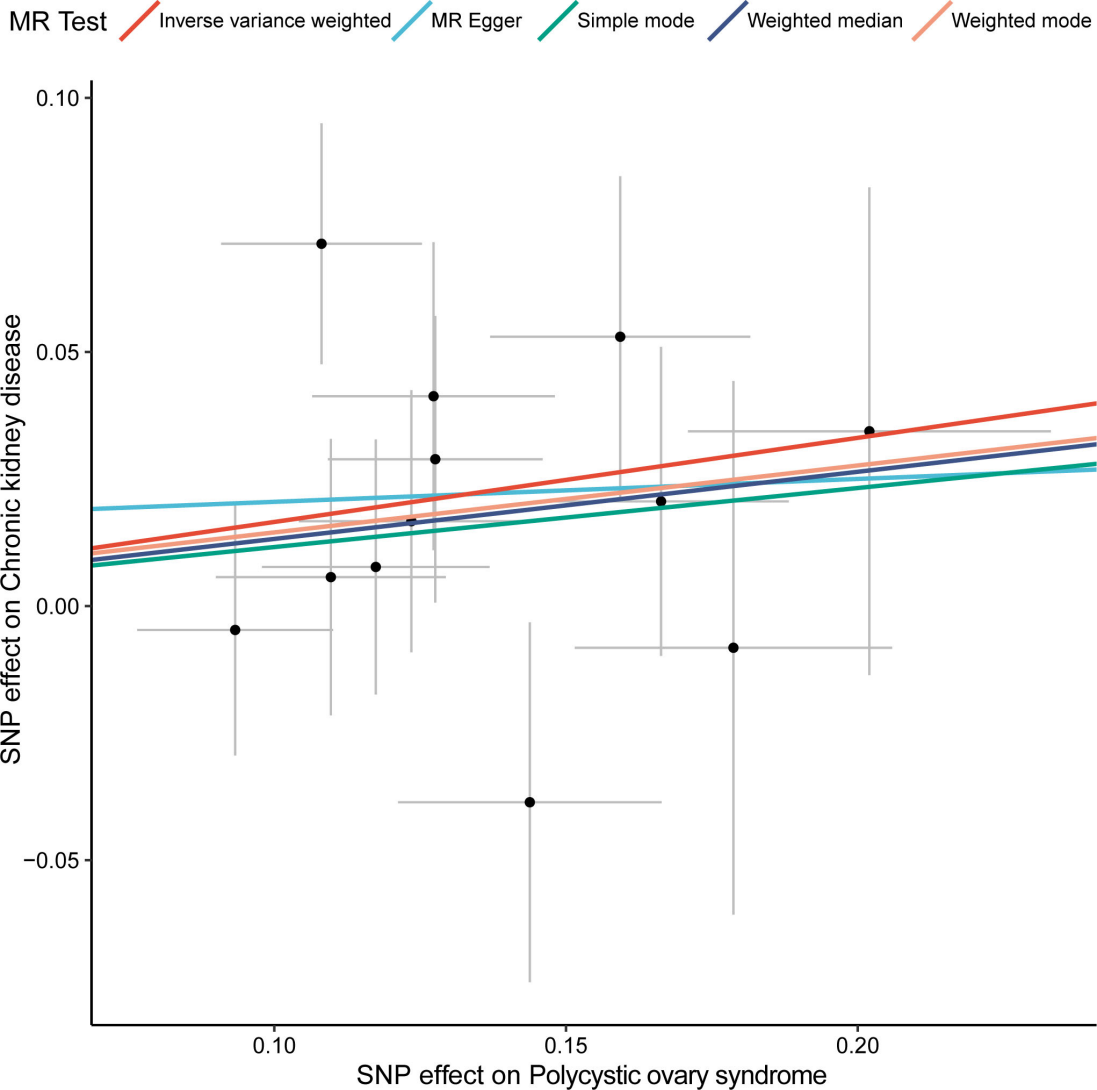
Two-sample MR analysis of the effect of PCOS on CKD using different methods. X-axis represents the effect of SNP on exposure (polycystic ovary syndrome) and the Y-axis represents the effect of SNP on outcome (chronic kidney disease), and the colored lines indicate the results of fitting different MR methods. MR, Mendelian randomization; SNP, single-nucleotide polymorphism.

We first used the selected 12 SNPs as the instrumental variables to assess the association between CKD and PCOS. The inverse variance weighted (IVW) method was used for the MR analysis, while the other four methods (MR Egger, Simple mode, Weighted median and Weighted mode) were applied to confirm the robustness of the results (**Figure 1**). In the IVW analysis, the effect odds ratio (OR) of PCOS on CKD was 1.180 (95% CI, 1.038-1.342; P = 0.010) (**Table 1**), which positive association exists between PCOS and CKD. MR-Egger do not detect potential horizontal pleiotropy for PCOS (P = 0.726), and there was no significant heterogeneity detected through Cochran’s Q test with both MR-Egger and IVW methods (P = 0.361, P = 0.435, respectively).

**Table 1:** The causal effect between PCOS and CKD.

Exposure	Outcome	Methods	nSNP	SE	OR (95%CI)	P value
Polycystic ovary syndrome	Chronic kidney disease	MR Egger	12	0.341	1.046(0.536,2.042)	0.898
		Weighted median	12	0.086	1.141(0.963,1.352)	0.127
		Inverse variance weighted	12	0.065	1.180(1.038,1.342)	0.011
		Simple mode	12	0.146	1.123(0.844,1.495)	0.443
		Weighted mode	12	0.131	1.141(0.882,1.475)	0.337

SNP, single-nucleotide polymorphism; SE, standard error; OR, odds ratio; 95%CI, 95% confidence interval.

### No causal relationship between PCOS and CKD risk factors

The occurrence of CKD is associated with many risk factors. We wondered whether PCOS could contribute to the occurrence of CKD by enhancing the risk factors of CKD. Several common CKD risk factors were selected and analyzed by multiple methods (MR Egger, WM, IVW), and none of them were statistically significant. The beta value and p value are as follows: Type 2 diabetes (0.964, 95% CI, -0.908-1.024; P = 0.235), Autoimmune diseases (1.012, 95% CI, 0.952-1.076; P = 0.704), Cystic kidney disease (1.089, 95% CI, 0.790-1.500; P = 0.604), Obesity class 1 (defined as BMI ≥ 30 kg/m2, -0.979, 95% CI, -0.906-1.058; P = 0.594), Obesity class 2 (defined as BMI ≥ 35 kg/m2, -0.904, 95% CI, -0.798-1.025; P = 0.117), Obesity class 3 (defined as BMI ≥ 40 kg/m2, -0.940, 95% CI, 0.736-1.200; P = 0.618), Hypertension (0.999, 95% CI, 0.994-1.004; P = 0.585) (**Supplementary Figure 1**). Significant heterogeneity was observed between hypertension and PCOS (IVW analysis: Q = 21.780, P=0.026). No horizontal pleiotropy was identified by MR-Egger test.

### PCOS affects the changes of fibroblast growth factor 23, creatinine and cystatin C of CKD

**Figure 2:**
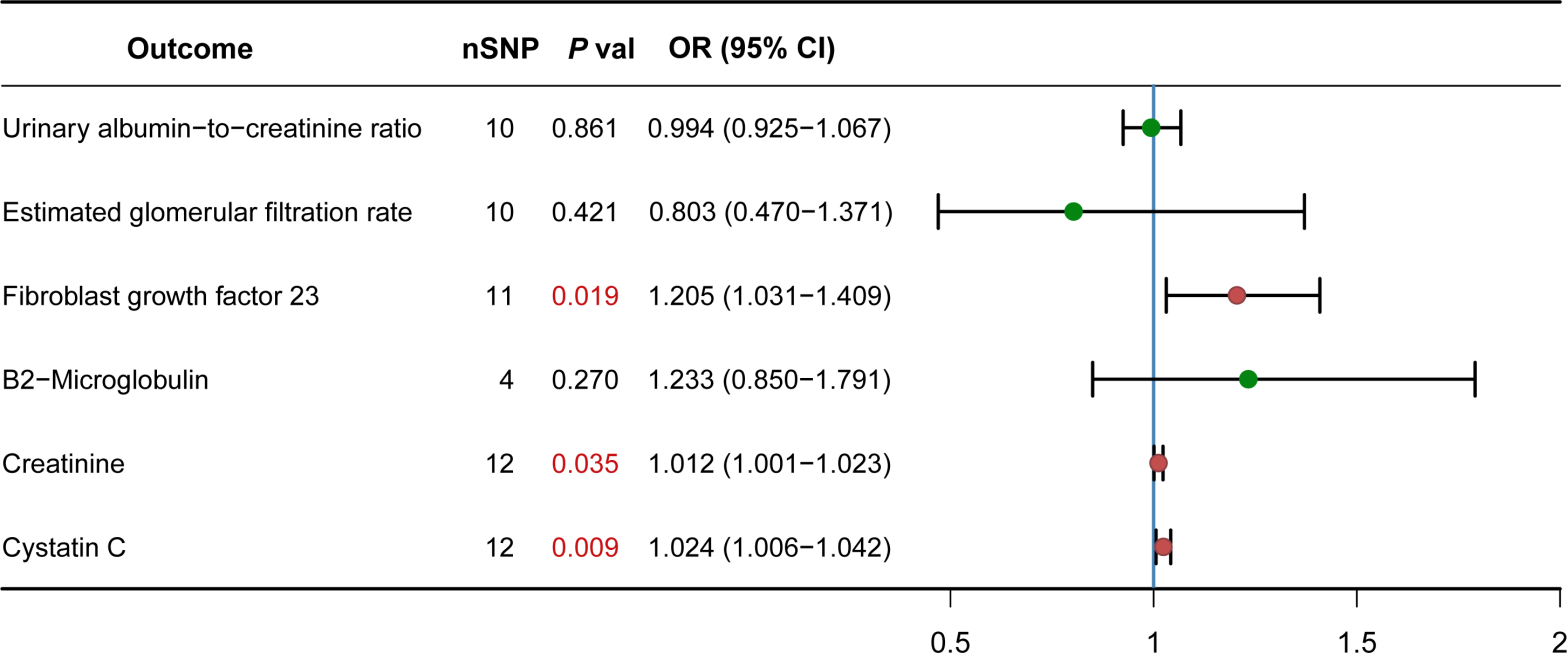
Causal effect of PCOS on CKD Serological indicators. Forest plots showing the range of OR values for different serological indicators. The green point (p>0.05) and red point (p<0.05) represents OR values of each CKD serological indicators, the vertical lines on either side of the point represent the 95% confidence interval. OR, odds ratio; 95%CI, 95% confidence interval.

Serological indicators are generally used to diagnose diseases or reflect the severity of diseases. We want to know whether there is a causal relationship between some serological indicators of CKD and PCOS. The IVW analysis showed that PCOS and some indicators have causal relationship such as fibroblast growth factor 23 (1.205, 95% CI, 1.031-1.409; P = 0.019), creatinine (0.012, 95% CI, 1.001-1.023; P = 0.035), cystatin C (1.024, 95% CI, 1.006-1.042; P = 0.009). There are no relationships between PCOS and other serological indicators: estimated glomerular filtration rate (-0.803, 95% CI, 0.470-1.371; P = 0.421), β2-microglobulin (1.233, 95% CI, 0.850-1.791; P = 0.270), urinary albumin-to-creatinine ratio (0.994, 95% CI, 0.925-1.067; P = 0.861) (**Figure 2**). The heterogeneity was observed between PCOS and serological indicators for CKD by the IVW analysis: fibroblast growth factor 23 (Q = 5.593, P= 0.848), creatinine (Q =12.209, P = 0.348), cystatin C (Q = 24.178, P= 0.012), estimated glomerular filtration rate (Q = 14.978, P= 0.092), β2-microglobulin (Q =1.172, P = 0.760), urinary albumin-to-creatinine ratio (Q = 33.101, P<0.001). For the horizontal pleiotropy, we found that there was no evidence between PCOS and serological indicators of CKD: fibroblast growth factor 23 (P= 0.971), creatinine (P = 0.212), cystatin C (P= 0.357), estimated glomerular filtration rate (P= 0.180), β2-microglobulin (P = 0.600), except for urinary albumin-to-creatinine ratio (P= 0.046).

### No causal association between PCOS and renal tubule injury biomarkers

An increasing number of renal tubule injury biomarkers are being discovered and applied in the diagnosis and treatment of CKD. However, whether there should be a relationship between PCOS and renal tubule injury biomarkers still remains a mystery. We next performed IVW analyses for PCOS and those biomarkers. The result shows that there was no causal relationship between the two: insulin-like growth factor-binding protein 7 levels (0.918, 95% CI, 0.741-1.135; P = 0.429), epidermal growth factor levels (0.978, 95% CI, 0.902-1.060; P = 0.588), monocyte chemoattractant protein-1 levels (1.036, 95% CI, 0.959-1.118; P = 0.368), tumor necrosis factor receptor 1 levels (1.035, 95% CI, -0.970-1.104; P = 0.301), tumor necrosis factor receptor 2 levels (1.063, 95% CI, 0.974-1.160; P = 0.173), chitinase-3-like protein 1 levels (1.014, 95% CI, 0.944-1.088; P = 0.712), kidney injury molecule 1 levels (1.006, 95% CI, -0.937-1.080; P = 0.868), neutrophil gelatinase-associated lipocalin (1.033, 95% CI, 0.880-1.214; P = 0.688), interleukin 6 (1.118, 95% CI, 0.956-1.306; P = 0.163), interleukin 18 (1.042, 95% CI, 0.854-1.270; P = 0.688), interleukin 10 (0.985, 95% CI, 0.578-1.681; P = 0.957) (**Supplementary Figure 2**). For the heterogeneity, we found that monocyte chemoattractant protein-1 levels (IVW: Q = 25.601, P= 0.007), tumor necrosis factor receptor 2 levels (IVW: Q = 34.016, P< 0.001), chitinase-3-like protein 1 levels (IVW: Q = 22.586, P= 0.020) were statistically significant. MR-Egger do not detect potential horizontal pleiotropy.

## Discussion

Previous researches have demonstrated that comorbidities related to PCOS included obesity, type 2 diabetes, cardiovascular disease, etc., but the relationship between PCOS and CKD is still contradictory (32–34). Patil et al. showed that women with PCOS may be at increased risk for development of CKD with advanced age (32). Behboudi-Gandevan et al. found that the risk of CKD in patients with PCOS was like the common female population, and larger studies with long-term follow-up was needed (33). Since there is no consensus, this study used Mendelian randomization to explore the potential casual association of PCOS and CKD. Our result suggested that the association between PCOS and CKD was statistically significant when using IVW analysis, and the trend of other sensitivity analyses were consistent with the results of IVW, which indicated that the causal relationship between PCOS and CKD we obtained is relatively robust. Our findings indicated that PCOS is causally linked to CKD. To learn more about the role and impact of PCOS in CKD development, the risk factors were evolved as outcomes and MR analysis were conducted (26). Several studies suggest that PCOS causes CKD because of metabolic-related factors such as obesity and disordered hormone secretion (32, 33). However, our research did not find the causal relationship between PCOS and CKD risk factors. Future studies with larger sample sizes need to be performed to clarify the relationship between the two.

The clinical diagnosis of CKD mainly relies on the classical definition: estimated glomerular filtration rate (eGFR) <60 ml/min/1.73m^2^, or markers of kidney damage, such as albuminuria, hematuria or abnormal structure on imaging, persistent for at least 3 months (15). With the deepening of research on CKD diagnosis and treatments, other indicators were validly used in fundamental and clinical research. Elevated levels of urinary albumin-to-creatinine ratio often indicates glomerular injury. Previous reports showed the high level of urinary albumin-to-creatinine ratio in patients with PCOS (35).β2-microglobulin is another early and sensitive diagnostic indicator of kidney disease. However, our results suggested no causal relationship between PCOS and those indicators mentioned above (eGFR, urinary albumin-to-creatinine ratio and β2-microglobulin). Creatinine was used as an important indicator for CKD patients, and it also formed a collaboration equation for estimating glomerular filtration rate (36). New eGFR equations that incorporate creatinine and cystatin C (37) could provide more accurate information for diagnosing CKD. Thus, we also studied Cystatin C (Cys C), another novel predictor for the development of CKD (38). Cys C has been proposed as a potential glomerular filtration rate (GFR) marker for the early detection of CKD and skin interstitial fluid (ISF) Cys C equipment has been developed (39). Cystatin-C levels was associated with an increase in IL-6 and a decrease in SOD in PCOS patients. This result suggested immune dysregulation exist in PCOS patients may affect the function of other organs (40). Fibroblast growth factor 23 (FGF23), a bone-derived phosphaturic hormone for regulating 1,25-dihydroxyvitamin D3 (calcitriol), could decrease the number of sodium-phosphate cotransporter 2a (NaPi-2a) on the basolateral membrane of proximal tubule cells. In recent years, the role of FGF23 in CKD has gradually been discovered. Makoto Kuro-o considered that FGF23 increase is deemed necessary to compensate for the decrease in the nephron number during CKD progression (41). Previously studies have reported that FGF23 increasing, calcitriol decreasing, and parathyroid hormone increasing occur in this order during CKD progression (41). Another research suggested that decreased FGF23 could improve outcomes in CKD (42). Anyhow, FGF23 has been served as a marker and potential pathogenic factor for CKD progression (41). We performed MR analysis to determine whether PCOS influenced those indicators of CKD. These results showed that there might be a causal relationship between PCOS and those three indicators including creatinine, Cys C and FGF23 which hint a possible association between PCOS and CKD.

Renal tubular damage is the important pathological process leading from CKD to the end stage of various renal disease (43). We wondered whether PCOS might be directly targeting the renal tubular. We selected reported renal tubular injury markers to explore whether PCOS is causally related to them (27). The results showed that PCOS could not cause the changes of these biomarkers, which might imply that CKD induced by PCOS is not mediated by these biomarkers of renal tubular injury.

Overall, Mendelian randomization (MR) is a powerful tool in investigating the relationship between exposures and outcomes, it could help prioritize potential causal relationships. The advantage of MR is that confounding factors could be controlled, and the wastes of manpower and material resources could be overcome comparing with the randomized controlled trial. In addition, MR studies are generally considered to provide higher-quality evidence than observational studies. Our results suggested that PCOS can lead to CKD, might be an independent causal relationship. Nevertheless, the study also has certain limitations that must be acknowledged. The sample size is a limiting factor in such studies. The larger the sample size, the more reliable the conclusion. Gender factor is a possible bias in the current study because the CKD data included data on mixed-gender loci, but the PCOS data included only female data. This may have influenced the results to some extent, but it is difficult to isolate female CKD data alone. For further study, a long-term cohort stratified by age and sex is needed to validate our conclusions. In addition, our study sample was all European ancestry, so the generalizability of our results to other races/ethnicities is uncertain.

This is the first study to investigate the causal relation of PCOS and CKD according to our knowledge. In conclusion, our results revealed that there is a causal relationship between PCOS and CKD, but whether this causal relationship is direct or indirect is still unclear, large-scale prospective cohort studies are needed to validate the preliminary findings of our study, and even more basic experimental research need to be done with human tissue, to further clarify the deep molecular mechanisms. The clinical significance of this study is that in addition to the prevention of cardiovascular and diabetes complications in patients with PCOS, more attentions should also be paid to the long-term follow-up of renal function to provide an opportunity for early intervention to slow down the progression of CKD.

## Data availability statement

The original contributions presented in the study are included in the article/**Supplementary Material**. Further inquiries can be directed to the corresponding authors.

## Author contributions

YD performed analyses and wrote the manuscript. SL, LD, and FL helped to analyze the results and edited the manuscript. ML guided the study and edited the manuscript. All authors contributed to the article and approved the submitted version. 
