# Supplementary material for: Causal relationship between polycystic ovary syndrome and chronic kidney disease: A Mendelian randomization study
Source: Front Endocrinol (Lausanne). 2023 Mar 15;14:1120119. doi: 10.3389/fendo.2023.1120119 (PMC10050750; doi:10.3389/fendo.2023.1120119)
Supplement: Supplementary file 1 [file DataSheet_1.docx]

Supplementary Material

Causal relationship between Polycystic Ovary Syndrome and Chronic Kidney Disease：A Mendelian Randomization Study

**Yufei Du, Fengao Li, Shiwei Li, Li Ding*, Ming Liu***

Department of Endocrinology and Metabolism, Tianjin Medical University General Hospital, Tianjin, 300052, China

*** Correspondence:**

Ming Liu: [mingliu@tmu.edu.cn](mailto:mingliu@tmu.edu.cn), ORCID: 0000-0003-2665-4072;

Li Ding: dinglitml@tmu.edu.cn

# Supplementary Figures and Tables

## Supplementary Figures
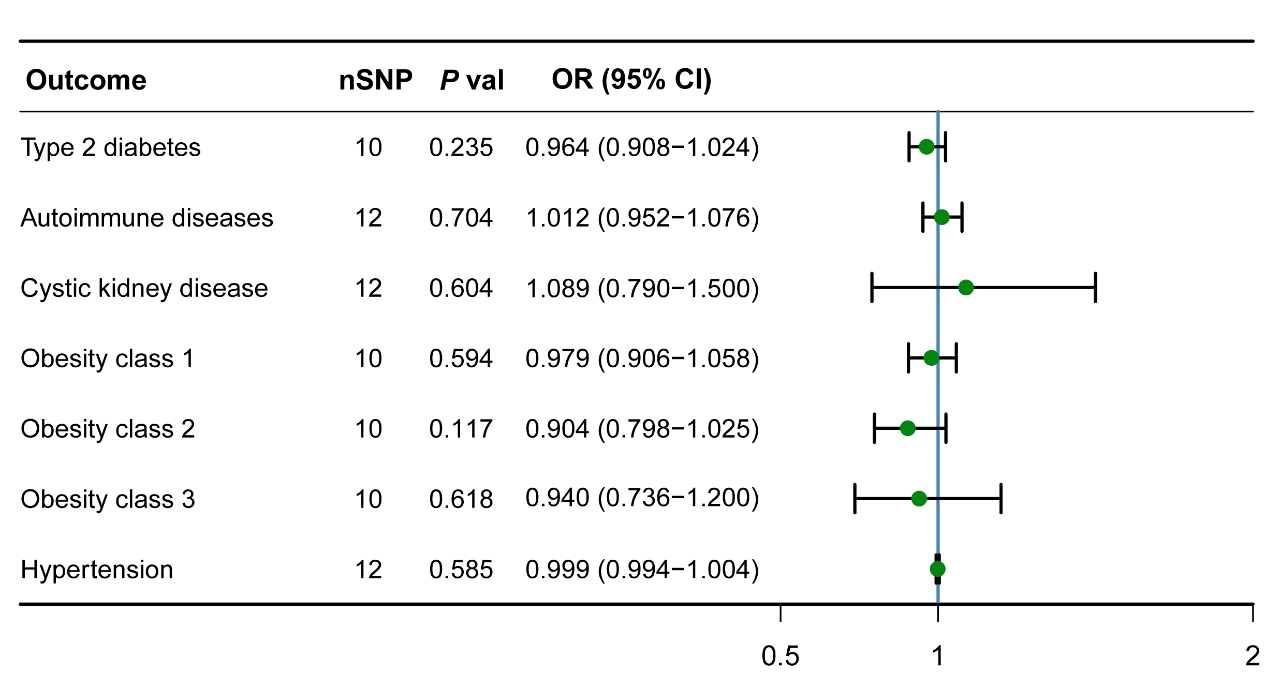


**Supplementary Figure 1.** Causal effect of PCOS on CKD risk factors. Forest plots showing the range of OR values for different risk factors. The green point represents OR values of each CKD risk factor, the vertical lines on either side of the point represent the 95% confidence interval. OR odds ratio, CI 95 95% confidence interval.


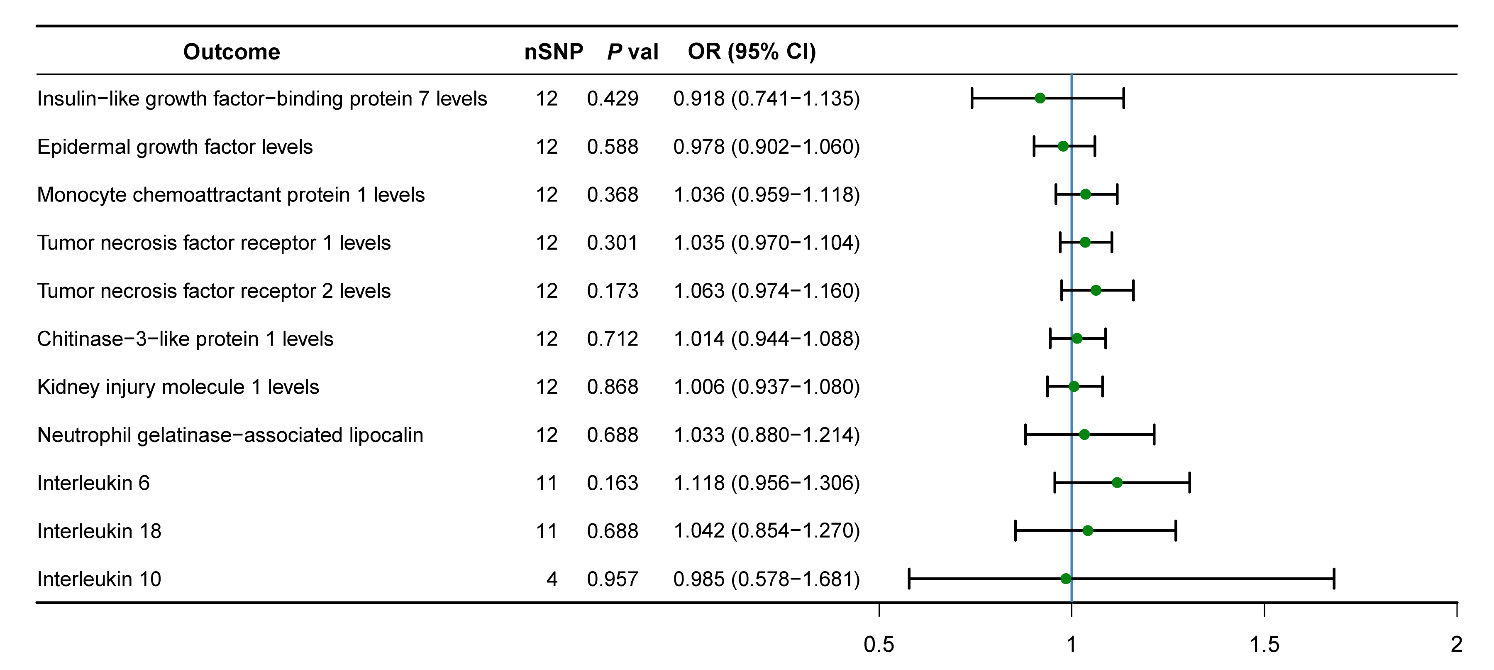
**Supplementary Figure 2.** Causal effect of PCOS on renal tubule injury biomarkers. Forest plots showing the range of OR values for different renal tubule injury biomarkers. The green point represents OR values of each renal tubule injury biomarker, the vertical lines on either side of the point represent the 95% confidence interval. OR, odds ratio, 95%CI, 95% confidence interval.

## Supplementary Table

| SNP | Effect Allele | Other Allele | EAF | Gene | Beta | SE | P Value |
| --- | --- | --- | --- | --- | --- | --- | --- |
| rs7563201 | A | G | 0.45 | THADA | -0.1081 | 0.0172 | 3.68E-10 |
| rs2178575 | A | G | 0.15 | ERBB4 | 0.1663 | 0.0219 | 3.34E-14 |
| rs13164856 | T | C | 0.73 | IRF1/RAD50 | 0.1235 | 0.0193 | 1.45E-10 |
| rs804279 | A | T | 0.26 | GATA4/NEIL2 | 0.1276 | 0.0184 | 3.76E-12 |
| rs10739076 | A | C | 0.31 | PLGRKT | 0.1097 | 0.0197 | 2.51E-08 |
| rs7864171 | A | G | 0.43 | C9orf3 | -0.0933 | 0.0168 | 2.95E-08 |
| rs9696009 | A | G | 0.07 | DENND1A | 0.202 | 0.0311 | 7.96E-11 |
| rs11031005 | T | C | 0.85 | ARL14EP/FSHB | -0.1593 | 0.0223 | 8.66E-13 |
| rs11225154 | A | G | 0.09 | YAP1 | 0.1787 | 0.0272 | 5.44E-11 |
| rs1784692 | T | C | 0.82 | ZBTB16 | 0.1438 | 0.0226 | 1.88E-10 |
| rs1795379 | T | C | 0.24 | KRR1 | -0.1174 | 0.0195 | 1.81E-09 |
| rs8043701 | A | T | 0.82 | TOX3 | -0.1273 | 0.0208 | 9.61E-10 |

SNP, single nucleotide polymorphism; EAF, effect allele frequency; SE, standard error

**Supplementary table 1.** SNPs from a large-scale genome-wide meta-analysis of polycystic ovary syndrome
